# Pregnancy-Related Extracellular Vesicles Revisited

**DOI:** 10.3390/ijms22083904

**Published:** 2021-04-09

**Authors:** Carmen Elena Condrat, Valentin Nicolae Varlas, Florentina Duică, Panagiotis Antoniadis, Cezara Alina Danila, Dragos Cretoiu, Nicolae Suciu, Sanda Maria Crețoiu, Silviu Cristian Voinea

**Affiliations:** 1 Department of Obstetrics and Gynecology, Polizu Clinical Hospital, Carol Davila University of Medicine and Pharmacy, 8 Eroii Sanitari Blvd., 050474 Bucharest, Romania; drcarmencondrat@gmail.com; 2 Alessandrescu-Rusescu National Institute for Mother and Child Health, Fetal Medicine Excellence Research Center, 020395 Bucharest, Romania; flory_duica@yahoo.com (F.D.); danilacezaraalina@gmail.com (C.A.D.); dragos@cretoiu.ro (D.C.); nsuciu54@yahoo.com (N.S.); 3 Department of Obstetrics and Gynecology, Filantropia Clinical Hospital, Carol Davila University of Medicine and Pharmacy, 011171 Bucharest, Romania; 4 Division of Molecular Diagnostics and Biotechnology, Antisel RO SRL, 024095 Bucharest, Romania; panosant89@gmail.com; 5 Department of Cell and Molecular Biology and Histology, Carol Davila University of Medicine and Pharmacy, 8 Eroii Sanitari Blvd., 050474 Bucharest, Romania; 6 Division of Obstetrics, Gynecology and Neonatology, Carol Davila University of Medicine and Pharmacy, 8 Eroii Sanitari Blvd., 050474 Bucharest, Romania; 7 Department of Obstetrics and Gynecology, Polizu Clinical Hospital, Alessandrescu-Rusescu National Institute for Mother and Child Health, 020395 Bucharest, Romania; 8 Department of Surgical Oncology, Prof. Dr. Alexandru Trestioreanu Oncology Institute, Carol Davila University of Medicine and Pharmacy, 252 Fundeni Rd., 022328 Bucharest, Romania; dr.voineasilviu@gmail.com

**Keywords:** extracellular vesicles, placenta, gestation, pregnancy disorders, liquid biopsy

## Abstract

Extracellular vesicles (EVs) are small vesicles ranging from 20–200 nm to 10 μm in diameter that are discharged and taken in by many different types of cells. Depending on the nature and quantity of their content—which generally includes proteins, lipids as well as microRNAs (miRNAs), messenger-RNA (mRNA), and DNA—these particles can bring about functional modifications in the receiving cells. During pregnancy, placenta and/or fetal-derived EVs have recently been isolated, eliciting interest in discovering their clinical significance. To date, various studies have associated variations in the circulating levels of maternal and fetal EVs and their contents, with complications including gestational diabetes and preeclampsia, ultimately leading to adverse pregnancy outcomes. Furthermore, EVs have also been identified as messengers and important players in viral infections during pregnancy, as well as in various congenital malformations. Their presence can be detected in the maternal blood from the first trimester and their level increases towards term, thus acting as liquid biopsies that give invaluable insight into the status of the feto-placental unit. However, their exact roles in the metabolic and vascular adaptations associated with physiological and pathological pregnancy is still under investigation. Analyzing peer-reviewed journal articles available in online databases, the purpose of this review is to synthesize current knowledge regarding the utility of quantification of pregnancy related EVs in general and placental EVs in particular as non-invasive evidence of placental dysfunction and adverse pregnancy outcomes, and to develop the current understanding of these particles and their applicability in clinical practice.

## 1. Introduction

Pregnancy, an efficiently regulated physiological process by which women give birth to offspring, is characterized by numerous adaptive changes—including, among others, anatomical, hormonal, metabolic, immunological and cardiovascular adjustments. Perhaps the most substantial, changes in the endocrine system help ensure the proper development of the growing fetus, particularly with the aid of the fetoplacental unit, which acts both as a meaningful hormone source and an efficient tissue barrier [1].

While most pregnancies progress smoothly, culminating in successful delivery, the wellbeing of the mother and/or the fetus can be affected by various abnormalities occurring during gestation. The most common pregnancy complications refer to gestational hypertension, gestational diabetes mellitus, maternal systemic inflammation, infections, premature delivery, and fetal growth restriction [2,3,4]. Additionally, the physiological evolution of pregnancy can also be adversely influenced by congenital anomalies occurring during intrauterine life, such as structural chromosomal abnormalities, heart defects, and neural tube defects [5,6,7]. Furthermore, these complications not only increase the odds of adverse pregnancy outcomes, but also impact the later development of the newborn, and may result in various maternal afflictions following parturition, such as hypertension or diabetes [8,9,10]. At present, the diagnosis of these conditions mainly relies on hematological tests and ultrasound screening, routine blood pressure monitoring and proteinuria tests for hypertension and pre-eclampsia, along with blood glucose and fasting blood glucose levels measuring for gestational diabetes [11]. While repeatedly proven reliable, it is not rare that, by these means, anomalies are not detected in the optimal timeframe for ensuring favorable outcome following clinical intervention. Therefore, the development and use of novel non-invasive biomarkers for timely diagnosis of pregnancy-related complications and/or fetal anomalies is critical in the current setting of increased perinatal morbidity and mortality associated with pregnancy complications. To this extent, the quickly emerging field of extracellular vesicle (EV) research holds strong evidence for the use of its components as non-invasive, accurate biological signatures.

Extracellular vesicles are cell-derived particles sheathed in a lipid bilayer that are naturally secreted into the extracellular space [12]. Though their functions often overlap, various subtypes have been suggested, with the three main established categories consisting of exosomes, ectosomes or microvesicles, and apoptotic bodies [13]. Exosomes vary in size, typically ranging from 30 to 150 nm in diameter [14,15], and are generated by the inward expansion of the endosome membrane, leading to the formation of multivesicular bodies (MVBs) rich in intraluminal vesicles (ILVs). When the MVB binds to the plasma membrane, ILVs are discharged in the form of exosomes [16,17]. Ectosomes, on the other hand, are commonly larger in size, reaching up to 1 μm in diameter [14,18,19], and are formed by the outward bulging of the plasma membrane, with the aid of cytoskeletal filaments [20,21]. Apoptotic bodies have been reported to reach up to 5000 nm in size [22], occurring as a result of cellular death accompanied by structural changes such as contraction and apoptotic blebbing [23]. The formation and discharge of MVBs and exosomes take place under the strict control of the endosomal sorting complexes required for transport (ESCRT) proteins [24,25,26], and are thought to be facilitated by certain growth factors [27]. Apart from ESCRT proteins, EVs also contain a group of marker proteins with no relation to the origin cell, including programmed cell death 6-interacting protein (PDCD6IP/Alix), tumor susceptibility gene 101 (TSG101), heat shock cognate protein 70 (HSC70), heat shock protein 90β (HSP90β), tetraspanin 28 (TSPAN28/CD81), tetraspanin 29 (TSPAN29/CD9), and tetraspanin 30 (TSPAN30/CD63) [28,29]. Tetraspanins are membrane proteins containing four transmembrane domains that play important roles in the fabrication and biosynthesis of EVs [30,31]. Further on, within their cargo, they also carry certain bioactive lipids and prostaglandins [32], along with RNA in the form of messenger RNA (mRNA), microRNA (miRNA), and long non-coding RNA (lncRNA) [33], and interestingly, little or no DNA. Ectosomes, on the other hand, differentiate themselves by markers such as annexin V, selectin, membrane type 1-matrix metalloproteinase (MT1-MMP), CD40, and flotillin-2 [19,34], while apoptotic bodies are rich in DNA fragments, intact organelles, histones, and annexin V [21,35].

The International Society for Extracellular Vesicles (ISEV) currently recommends the use of the generic term ‘extracellular vesicle’, since establishing the exact biogenesis of the discussed particle is a difficult task. Furthermore, it is also suggested that authors rather describe the size, biochemical composition and origin of the EV, rather than erroneously attributing it a subtype [17]. Additionally, in order to confirm the presence of EVs, ISEV recommends identifying the presence of three categories of markers common to all EVs, as highlighted in Table 1.

Due to high degree of heterogeny of these molecules, numerous isolation and identification methods have been developed, including labeling technologies such as flow cytometry, immunoelectron microscopy, or western blot for specific markers [36,37]. Some of the most common isolation and detection approaches used to evaluate the pregnancy-related nanovesicles have been summarized in Table 2 and Table 3, along with some of their advantages and disadvantages.

While initially regarded as debris, lacking any biological purpose, EVs have over time been demonstrated to play significant roles in intercellular communication, carrying both autocrine and paracrine functions following their secretion [52]. Additionally, due to their immune properties consisting of their behaving as antigen-presenting agents, exosomes especially have been shown to trigger immune responses [53,54]. Moreover, in the central nervous system, EVs have been reported to maintain the myelin coating and promote endogenous brain repair processes, thus making them valuable players in the post stroke recovery period [55,56]. In cancer disease, exosomes released by tumor cells can act as signal transduction mediators while facilitating not only neoplastic development, growth, and metastasis, but also chemoresistance [57,58]. However, above all, the most common interest in the field of EVs probably resides in their potential to serve as biomarkers due to their heterogeneous cargoes unique to specific conditions. To this extent, onco-hematological diseases such as acute myeloid leukemia can be diagnosed by identifying specific mutations in plasma EVs identical to those observed in leukemia cells [59,60]. The use of EVs as biomarkers in disorders of the central nervous system such as Alzheimer’s and Parkinson’s disease has also been reported [61,62], while accumulating evidence points towards a similar use of EVs in coronary artery disease [63,64]. Perhaps the most significant progress has, however, been made in cancer, with clinical trials already on the way, regarding their use as diagnostic tools and/or therapeutic instruments [65,66,67]. Still, more and more data supports the utilization of EVs as biomarkers in pregnancy-related complications, exosome analysis posing advantages such as the accessibility of blood sampling and detection in early pregnancy [11,52,68]. In this regard, a brief exemplification of the multitude of roles that EVs play in both normal and pregnancy-related disorders is summarized in Table 4, and further discussed in the following sections.

## 2. Extracellular Vesicles in Normal Pregnancy

In physiological pregnancy, EVs and exosomes in particular have time and again been indicated to act as components of the fetal-maternal communication during implantation and placentation [69,70,71], while also modulating the maternal immune response [72,73,74,75], maintaining cellular metabolic homeostasis [76,77,78], promoting fetal vasculogenesis together with maternal uterine vascular adaptation [79,80,81], and preparing the uterus for in the delivery process [82,83].

Attached to the wall of the uterus, the placenta constitutes the interface between the mother and fetus in the gestational period, ensuring gas exchange, nutrient and waste transfer, immunoglobulin transport, and hormone secretion. The maternal-fetal communication is possible either through simple or facilitated diffusion, active transport, or by means of EVs [45,84,85]. Through their content, embryonic EVs that are engulfed by specific maternal cells, end up regulating maternal adjustments. Further on, placental EVs aid the vascular changes brought about by the pregnancy, while also reflecting the placental function and fetal growth [86]. Placental-derived EVs distinguish themselves mainly through their positivity for the syncytiotrophoblast (STB) marker placental alkaline phosphatase (PLAP), among other STB-derived EVs (STBEVs) [37,86], while both early and term placental cytotrophoblast cells have been demonstrated to secrete, by means of exosomes, members of the B7 family of immunomodulatory molecules, namely B7-H1 (CD274), B7-H3 (CD276), and human leukocyte antigen-G5 molecules (HLA-G5) [87]. Maternal and fetal exosome transfer in both directions has been demonstrated using fluorescently labeled exosomes in pregnant mouse models, thus reinforcing the isolation of exosomes from maternal blood samples as a non-invasive liquid biopsy [85,88].

Similar to other EVs, placenta-derived EVs are abundant in miRNAs, regulators of gene expression at post-transcriptional level, exerting their effects by targeting multiple mRNAs [89]. By these means, miRNAs carried by EVs and transported to specific cells end up modifying the gene expression pattern of the recipient cells. Among placenta-associated miRNAs, 46 miRNAs belonging to the chromosome 19miRNA cluster (C19MC) have been identified, being expressed in villous trophoblasts [90,91]. Among these, miR-517b favors TNFα expression [92], while miR-516b-5p, miR-517-5p, and miR-518a-3p have been shown to impact the PI3K-Akt and the insulin signaling pathways, their expression levels being regulated by various stimuli, including oxidative stress and blood glucose levels [93]. Furthermore, in healthy dairy cow pregnancy models, placental exosome-derived miR-499 has been shown to downregulate NF-κB activation by targeting the Lin28B/let-7-ras signaling axis, therefore maintaining a slight proinflammatory profile [94].

### 2.1. EVs in Embryo Implantation

Embryo implantation, first in the series of events required for a prosperous pregnancy, is a crucial process necessitating a chain of molecular operations that ultimately accomplish the adhesion of the trophectoderm to the endometrial epithelial cells [95,96]. While mediators such as adhesion molecules, growth factors, hormones, and cytokines are known as crucial for endometrial receptivity, EVs have more recently been shown to aid the implantation process [97,98,99]. To this extent, it has been proposed and reported that endometrium-derived EVs are assimilated and internalized by both trophoblasts and surrounding endometrial cells, eventually enhancing their adhesive capacity, especially by means of gene expression modulation due to their content in miRNA [100]. In this regard, Ng YH, and colleagues have analyzed a panel of 227 endometrial exosomal miRNAs and showed that numerous of their target genes were in fact crucial for implantation, since they were responsible for regulating not only essential pathways, such as the VEGF pathway, the Toll-like receptor pathway and the Jak-STAT pathway, but also extracellular matrix (ECM)-receptor interactions and adherens junctions [100]. On the same note, Vilella et al. have shown that endometrium exosome-derived hsa-miR-30d, when taken up by trophoblasts, enhances the gene expression of Integrin Subunit Alpha 7 (Itg7), Integrin Subunit Beta-3 (Itgb3), and Cadherin 5 (Cdh5) proteins, all three required for blastocyst implantation [101], while Greening and colleagues have later demonstrated that endometrial EVs, when internalized by the trophectoderm, augment their adhesiveness via the focal adhesion kinase (FAK) signaling pathway [102].

### 2.2. EVs in Spiral Artery Remodeling

Following successful implantation, decidualization and placentation take place, the resulting placenta ensuring the necessary resources for the optimal development of the embryo [103]. The nutrient supply is facilitated by the uterine spiral arteries, which go through substantial transformation under the influence of adaptive mechanisms carried out by cellular and molecular factors [104]. Specifically, both cellular and extracellular components of the maternal uterine spiral arteries undergo modifications such as apoptosis, hyperplasia and hypertrophy, migration, and ECM remodeling, all under the rigorous coordination of invasive cytotrophoblast cells and decidual natural killer (NK) cells [105]. Trophoblast cells end up replacing the distal endothelial cells, acquiring a low-resistance vascular bed phenotype, fitting for unrestricted blood flow [106]. During placental development, the migration of vascular smooth muscle cells (VSMC) plays a key role in spiral artery remodeling, a movement which has been demonstrated to be in part promoted by EVs released by extravillous trophoblast (EVT) cells via a novel EVT-VSMC exosomal communication pathway [107]. Furthermore, exosomal miRNAs together with vascular endothelial growth factor A (VEGFA) have been reported to be discharged by the implanted embryo, so as to adjust blood flow [79,108]. Dependent upon oxygen levels, placental EVs have also been reported to stimulate vasculo-angiogenesis, especially in hypoxic conditions [71]. On the same note, Jia and colleagues have researched the role of maternal and umbilical cord blood exosomes on angiogenesis. By analyzing healthy pregnant women, they found that both maternal and umbilical exosomes promoted human umbilical vein endothelial cells (HUVEC) proliferation and migration, along with angiogenesis, with 258 miRNAs being upregulated in both types of exosomes [80]. Other trophoblast cells derived-EVs that have also been reported to have pro-angiogenic effects by enhancing the proliferation of maternal endothelial cells via particular angiogenesis-related miRNA regulation have been identified in umbilical cord blood [109].

### 2.3. EVs in Parturition

Provided that the course of pregnancy is not impaired, delivery is set to take place following fetal maturation, as a result of a parturition cascade involving proinflammatory events that ultimately trigger labor [110]. Still, the precise mechanism that leads to the initiation of the delivery process has not been completely elucidated, but EVs are thought to act as mediators that, mainly due to their content rich in complex molecules, end up reprogramming the phenotype of surrounding cells, eventually regulating their function [111]. In this regard, Menon and colleagues have analyzed placental EVs during pregnancy and at delivery, and found that samples at term associated a group of upregulated genes known to be regulators of epithelial mesenchymal transition (EMT). They hypothesized that, when approaching term, fetal components of the placenta undergo EMT, which leads to an increase in mesenchymal cells susceptible to oxidative stress followed by subsequent inflammation that precipitates delivery [112,113]. Moreover, as a response to increased oxidative stress, it has been demonstrated that phosphorylation of p38 mitogen-activated protein kinase (MAPK), an indicator of term parturition, takes place in amniotic epithelial cell (AEC)-derived exosomes [114]. Along the same lines, Hadley et al. later investigated whether oxidative stress prompted the production of exosomes by AEC, and found that indeed AEC undergoing oxidative stress released around seven times more exosomes than control cells, which, in turn, lead to the activation of the NF-κβ protein complex along with an increase in PGE2, IL-6, and IL-8 in endometrial and myometrial cells [115]. On a similar note, Sheller-Miller et al. have revealed that, predictably, maternal plasma EV concentration enhanced with gestational age, while EVs rich in particles promoting inflammation—e.g., plasminogen (PLG), catalase, TNF-α— were dominant ahead of parturition [82].

## 3. Systemic Inflammation in Pregnancy

### 3.1. Definition, Implications

Inflammation represents one of the many core processes involved in the success of every procreation endeavor. More than 2000 years have passed since Celsius coined the description of the inflammation’s cardinal signs, known as “rubor et tumor cum calore et dolore” (redness and swelling with heat and pain), and throughout history, extensive research has been conducted in order to decipher the true nature of this pathological expression in terms of cellular or molecular events [116,117]. Even though the definition of inflammation has suffered several adjustments over time, we recognize today that it should embrace not only the variability of the context in which is used but also the lens of detail related to molecular characterization, in order to avoid misinterpretations when it comes to defining the cascade of events [118]. In this regard, inflammation is viewed to date as a complex biological response of injured tissues to various traumatic agents, and can be described from a clinical, histopathological, and molecular standpoint. Outlining the clinical aspect of inflammation as increased local temperature and erythema due to vasodilation, tumescence due to increased vascular permeability and pain due to stimulation of nociceptors, was finally complete when Galen, Sydenham, and Virchow promulgated the notion of “functio laesa”, ultimately leading the way towards molecular exploration of this pathological event [119,120,121]. Major limitations have been uplifted while defining inflammation just on clinical terms, when pathologists characterized this process by a complex interplay of cellular interactions that evolve in successive and particular phases, that lead to healing. Consequently, the initiation of an inflammatory response translates into cellular release of molecules as a response to injury, that alter the vascular permeability, facilitating the passage of plasma, inflammatory cells such as neutrophils, platelets, cytokines, chemokines, and coagulation factors [122,123]. Following chemotaxis and diapedesis of specific elements such as macrophages into the injured tissue, the enzymatic digestion and phagocytosis begins, allowing the clearance of foreign material/infectious organisms and damaged tissue components, thus preparing the site of trauma to repair processes that ultimately and hopefully restore the normal biological constants and functions [124,125].

Inflammation and specifically sterile inflammation is a major regulator from early pregnancy to parturition [126]. During early pregnancy and labor, inflammation is important for blastocyst implantation and myometrial activation respectively, whereas its inactivation during placental development has a key role for the maternal tolerance of the paternal origin fetal antigens. Dysregulated inflammatory maternal responses can lead to a lot of morbid gestational conditions, such as fetal growth restriction [127], preterm birth [128], miscarriage [129], and preeclampsia [130]. Pattern recognition receptors (PRRs), such as Toll-like receptors 1–11 (TLRs) and NOD-like receptors, have the ability to induce inflammatory responses mediated by cytokines [131,132]. Thus, effector ligands for these receptors with damage associated molecular patterns (DAMPs) or pathogen associated molecular patterns (PAMs) can regulate the maternal inflammatory state during gestation and labor. DAMPs which have been also referred to as alarmins, such as cfDNA, uric acid, high-mobility group box 1 and interleukin-1α, can trigger inflammatory responses through PRRs, RAGE, and IL-1R [133,134,135,136]. The inflammatory response which has been attributed to cfDNA, is carried out though activation of TLR9, which is a PRR that likewise exerts its action after binding of unmethylated CpG fragments of bacterial or viral origin [137]. The activation of TLR9 can be performed as well by circulating mitochondrial DNA and cell free fetal DNA (cffDNA) [138]. As TLR9 is present inside the cytoplasm, its activation requires the endocytosis of cffDNA [139]. This procedure seems to be facilitated by placental syncytiotrophoblast microvesicles (SCTMs) which include cffDNA and get engulfed through phagocytosis by placental or circulating granulocytes. The increase of cffDNA has been implicated in normal pregnancies, as it significantly rises at the end of gestation during parturition, as well as in pathological conditions such as preeclampsia [140,141].

### 3.2. EVs in Inflammation

As the course of pregnancy is determined by the balance between pro-inflammatory and anti-inflammatory agents, the concentration and composition of placental EVs have a significant effect, due to their regulating roles in inflammation. As chronic systemic inflammation during pregnancy has been linked to various adverse outcomes, including premature rupture of membranes (PROM), neurodevelopmental delays and long-term brain impact [142,143], early treatment options ensured by timely detection could help reduce the overall impact of inflammation. For instance, since pre-eclampsia is knowingly associated with an immune imbalance characterized by an abundance in pro-inflammatory T cells cytokines coupled with diminished immunoregulatory agents [144], trophoblast-derived EVs have been found to be rich in high mobility group (HMG) nuclear proteins acting as endogenous danger signals reflecting cell and tissue damage [145]. Similarly, preeclamptic patients have been shown to carry low levels of miR-548c-5p both in placental mononuclear blood cells and serum EVs. The significance of this finding lies in the fact that miR-548c-5p is an established anti-inflammatory factor that targets the receptor-type tyrosine-protein phosphatase O (PTPRO) via the NF-κB signaling pathway, thus inhibiting macrophage activation and proliferation [146]. The proinflammatory state characteristic of preeclamptic patients can further be reflected by the upregulated exosomal miRNAs that encourage inflammatory responses, such as miR-155, involved in the IL-17A pathway [147], miR-494 which, by inhibiting prostaglandin E2 (PGE2), blocks the polarization of macrophages into an M2-type, promotors of inflammation resolution [148,149], miR-181a, regulator of the TGFβ pathway [150], and miR-210, which, by targeting the signal transducer and activator of transcription 6 (STAT6), leads to a decline in anti-inflammatory IL-4 secretion [151]. In a similar fashion, GDM patients have also been shown to associate a proinflammatory state, with GDM placenta-derived EVs rich in proinflammatory cytokines releasing significant amounts of tumor necrosis factor α (TNF-α), granulocyte macrophage colony-stimulating factor (GM-CSF), interferon-γ (IFN-γ), IL-6, and IL-8 [152]. Furthermore, high blood glucose levels have been shown to trigger the release of EVs from trophoblast cells which, in turn, increase the release of proinflammatory cytokines such as GM-CSF, IL-6. IL-8, IL-10, and TNF-α, from endothelial cells [153,154]. During pregnancy, the inflammatory immune response is additionally promoted by infection, be it intrauterine, referring to abnormal genital tract colonization [155], or remote, including, among others, periodontitis, and urinary tract infections [142,156]. Moreover, factors such as low socioeconomic status, poor diet, high levels of stress can also elicit maternal inflammatory responses expressed by elevated IL-6 levels [143], that do not necessarily culminate in preterm birth [157], but rather in altered neurodevelopment in infants, ascertained by decreased working memory performance as an aspect of impaired executive function [143,158,159].

## 4. Gestational Hypertension

### 4.1. Definition, Etiology, Epidemiology

Hypertension is a common morbidity among women of reproductive age (15–49 years) in modern societies, having a prevalence of around 8% in the United States [160]. During pregnancy, hypertensive disorders of different severity levels have been described—such as gestational hypertension, preeclampsia, and eclampsia—which can confer health complications to the mother and the child, prepartum as well as postpartum. The diagnostic criteria for hypertension in pregnancy, as set by different organizations, are similar and stratify hypertension according to its severity (Table 5). Gestational hypertension is defined as a maternal first-time diagnosis of hypertension after 20 weeks of gestation, which resolves postpartum. On the other hand, preeclampsia is defined as a gestational hypertension accompanied by proteinuria or end-organ damage, such as kidney or liver injury, uteroplacental dysfunction, hematological abnormalities, or neurological complications. These neurological complications can include grand mal seizures, which are referred to as eclampsia [8].

Preeclampsia can be triggered by hypertension in pregnancy, as it has been estimated to emerge in 35% and 25% of women with gestational hypertension or chronic hypertension respectively [165,166]. Moreover, risk factors are considered the genetic predisposition, such as mutations in the fms related receptor tyrosine kinase 1 (FLT1) gene and trisomy 13, immunological abnormalities, such as imbalance between decidual natural killers and regulatory T cells, maternal comorbidities—such as diabetes, obesity, and chronic kidney disease, in vitro fertilization, as well as older maternal age [167,168]. The pathogenesis of preeclampsia can be separated into two stages, including aberrant placental perfusion and placental dysfunction during the first stage and systemic endothelial dysfunction of the vessels that leads to the development of the maternal syndrome during the second stage [169]. In Stage I, which occurs during the first and second trimesters, an ineffective invasion of uterine spiral arteries induces placental hypoxia, endoplasmic reticulum dysfunction and oxidative stress that triggers a cascade of inflammatory events. In Stage II, which occurs during the third trimester, an imbalance is observed between angiogenic and anti-angiogenic factors, while inflammatory mediators are released. Angiogenic factors such as vascular endothelial growth factor (VEGF) and placental growth factor (PIGF) are downregulated; while anti-angiogenic factors such as soluble fms-like tyrosine kinase 1 (sFLT1), and soluble endoglin (sENG) are upregulated [169,170]. The induced systemic vascular dysfunction gives rise to the clinical manifestations of preeclampsia as well as eclampsia when cerebral oedema is produced [169]. Preeclampsia can be classified as early onset (EOPE), when it occurs before the 34th week of gestation, and late onset (LOPE), when it occurs after the 34th week. The development of EOPE has been attributed to the placental dysfunction and it has a higher possibility of being accompanied by intrauterine fetal growth restriction, whereas LOPE has been imputed to the outgrowth of placental circulatory system [171,172].

The burden of preeclampsia and eclampsia on public wealth is apparent and they are considered leading direct causes of maternal morbidity and mortality. On a worldwide scale, it has been estimated that they are the cause of around 50.000 maternal deaths annually [173]. Their prevalence differs around the world, having increased values in industrialized countries. In a study including data from NIS database (1,071,145 women), regarding patients with diagnosed preeclampsia or eclampsia between 2004–2012, a correlation has been found between the morbidity and/or mortality of the disorder and the racial descent of the women. In particular, an increased adjusted risk has been found for African American women compared to Hispanic or Caucasian women [174]. In Europe, the prevalence of preeclampsia has been estimated around 5.3% (95% CI: 1.8–9.3) and eclampsia 0.1% (95% CI: 0.0–0.4) [175].

### 4.2. EVs in Preeclampsia

During pregnancy, a major contributor for the maternal-fetal cell communication are placenta-derived EVs (PdEVs), which are released in the maternal circulation through the lysosomal pathway from the syncytiotrophoblast (STB) cells of the placenta. These particles contain genetic information, such as mRNA and miRNA molecules, which has been speculated to bear a diagnostic and prognostic value for pregnancy related morbidities, such as PE [176]. For the EVs identification, specific tetraspanins have been targeted in the past—such as CD63, CD70, CD81, and CD9—whereas for the identification of PdEVs, the PLAP protein is usually used [177,178]. PdEVs have been deemed to have an immunomodulatory role during placental development, through locally inducing apoptosis of the activated maternal lymphocytes which target placental paternal antigens [179]. Thus, PdEVs can promote the invasion of trophoblastic cells and serve towards maternal immunotolerance [180]. Nevertheless, aberrant production of PdEVs, such as their increased release in EOPE, can perturb the balance between Th1/Th2 and trigger a maternal systemic inflammatory response (MSIR) during stage II of PE [181].

Promising biomarkers of PE are the miRNAs which are enclosed in EVs. The elevated expression of exosomal hsa-miR-486-1-5p and hsa-miR-486-2-5p, irrespectively of gestational age, in pregnant women with PE could be used as an indication for early detection of the condition [182]. Moreover, in pregnant women with PE statistically significant difference has been found in the expression of the exosomal miRNAs hsa-miR-423-5p, hsa-miR-451a, hsa-miR-107, hsa-miR-15a-5p, hsa-miR-335-5p, hsa-miR-92a-2-3p, hsa-miR-103a-1-3p, hsa-miR-103a-2-3p, hsa-miR-92a-1-3p, and hsa-miR-126-3p [182]. The miRNAs miR-376c and miR-520g, which seem to have a significant role in cell migration and proliferation of the trophoblast cells, have been indicated as differentially expressed between pregnant women with PE and healthy ones [183,184].

## 5. Gestational Diabetes Mellitus

### 5.1. Definition, Etiology, Epidemiology

The hyperglycemic state that emerges in some pregnant women during the late second trimester or in the beginning of the next trimester has been referred in the past literature as gestational diabetes mellitus (GDM). This term was first mentioned and described by Carrington in 1957 [185]. Although GDM is more likely to develop after 24 weeks of gestation, it can exhibit its onset anytime during a pregnancy [186]. Based on the FIGO and WHO organizations, a hyperglycemic condition during pregnancy (HIP) can be characterized as GDM if it is a first-time diagnosis for a pregnant woman. On the other hand, if a pregnant woman has a previous diagnosis of diabetes or the HIP meets the criteria of WHO for diabetes, the condition is classified as diabetes in pregnancy (DIP) [187,188]. Nevertheless, the majority of HIP instances have been found to be classified as GDM [189]. The diagnostic criteria for GDM are not universally accepted, which is a factor that renders tough to compare the GDM prevalence on a global scale. Over the years and between different organizations, the diagnostic thresholds above which a hyperglycemia is considered overt diabetes, differ slightly. The most prominent criteria are the ones defined by IADPSG in 2010 [190] and adopted by WHO in 2013 [187] (Table 6).

**Figure 1:**
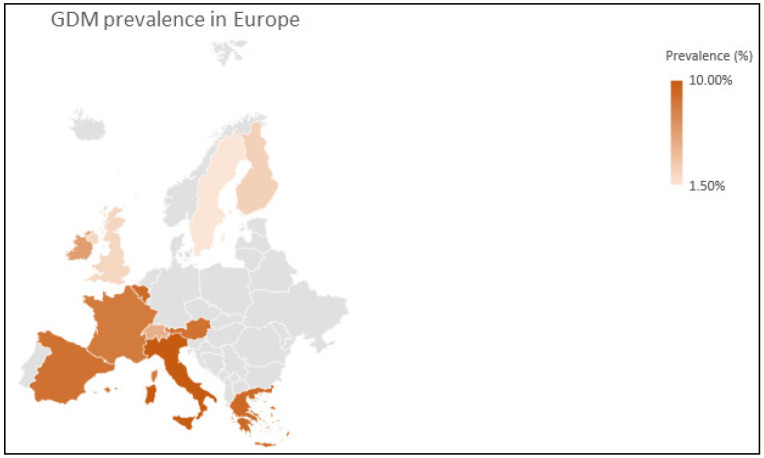
GDM prevalence in 11 European countries.

**Figure 2:**
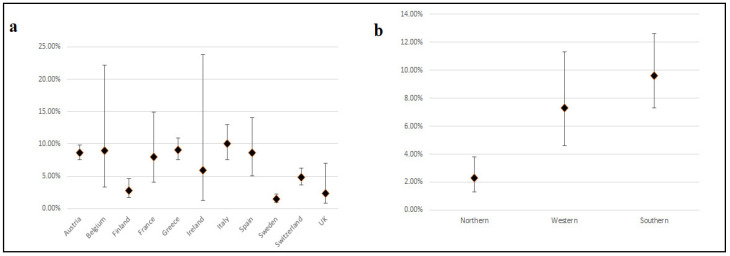
Mean GDM prevalence and 95% CI in: (**a**) 11 European countries; (**b**) Northern, Western, and Southern Europe.

Apart from hyperglycemia during pregnancy, GDM can be developed due to insulin resistance, which can be triggered by placental hormone production [194]. Among others, the GDM phenotype can be promoted by risk factors of pregnant women, such as older age, increased body mass index (BMI), personal or family history of diabetes, obesity, smoking, and polycystic ovary syndrome (PCOS). Although GDM is usually a transient disorder that recedes after pregnancy, it can confer an increased risk for GDM in subsequent pregnancies, while it also increases the possibility for the mother and the child to develop type 2 diabetes (T2D) in the future [195,196]. On top of that, ethnicity has been described as a significant factor for GDM prevalence [197]. The prevalence of GDM worldwide ranges between 1% and more than 30%, having regions or countries with significantly increased or decreased prevalence compared to the global mean. For instance, among the regions with high prevalence are countries of Middle East and North Africa, whereas Europe has the lowest prevalence values together with the highest variation across the continent [197]. According to data from the meta-analysis of Claire et al. [198], while the prevalence of GDM in Southern countries such as Italy, Greece and Spain is considerably high, in Northern countries such as Sweden and Finland is low (Figure 1 and Figure 2a). The mean prevalence values and the 95% CI between the Northern and Southern countries indicate a statistically significant difference between these regions of Europe (Figure 2b).

### 5.2. EVs in Gestational Diabetes Mellitus

The significance of EVs in pregnancy has been described is several studies, as their concentration increases in plasma during gestation [177,199]. Notably, pathologic conditions during pregnancy, such as GDM and PE, induce an increased concentration of placenta-derived EVs (PdEVs) in the circulation. The concentration of PdEVs has been found to be positively correlated with the maternal serum glucose levels, BMI and the weight of the fetus [200]. Increased PdEVs concentrations seem to trigger the proinflammatory state during pregnancy [76,201]. Moreover, an increased secretion of EVs from adipose tissue in GDM appear to be associated with the glucose metabolism of placenta and therefore it could be a regulating factor for the fetal growth [202]. These EVs seem to promote insulin resistance (IR) in obese women and contribute towards the development of GDM [154]. In parallel, the increased glucose levels in maternal circulation appear to induce the release of EVs from trophoblast cells and the secretion of proinflammatory cytokines from endothelial cells of the umbilical vein [153,203]. As described by Salomon et al. [152], the increased concentration in the circulation of PdEVs and total EVs could be used as indicative marker of GDM. Among the most interesting biomarkers of exosomes are the miRNAs, as in GDM cases specific miRNAs have been found to be associated with IR in skeletal muscle cells, namely hsa-miR-125a-3p, hsa-miR-99b-5p, hsa-miR-197-3p, hsa-miR-22-3p, and hsa-miR-224-5p [76]. Certain miRNAs have been implicated in the development of GDM [204], while miRNA such as mRNA-16-5p, -17-5p, and -20a-5p have been pointed out for their diagnostic significance in GDM [205]. Notably, miRNAs such as miR-222 from adipose tissue have been proposed as potential therapeutic targets [206].

## 6. Viral Infections during Pregnancy

### 6.1. Implications

It has been shown that viral infections occurring during pregnancy can induce a high risk of pregnancy loss by spontaneous abortion or fetal infection as an effect of subsequent congenital viral syndromes. In today’s practice, we face the lack of a real standard of prenatal care of viral infections during pregnancy except for a small range of pathogens known under the TORCH acronym, which include *Toxoplasma gondii*, Rubella virus, Cytomegalovirus (CMV), and Herpes simplex virus (HSV). While these guidelines allow doctors to early diagnose an infection, it is still unknown how to prevent adverse pregnancy effects [207,208]. A high predisposition to preterm labor or delivery is associated with viral infections. In consequence, understanding the higher risk to which pregnant women are subjected to is essential in designing an appropriate treatment or prevention plan [209]. In a developing fetus, the vertical infection can take place by trans- or paracellular transport from maternal blood into the fetal capillaries and over the villous trees, through the infected endothelial cells that spread to invasive the extravillous trophoblast, by ascension from the vagina, by transfer across the placental barrier of infected maternal immune cells or by breaches in the syncytiotrophoblast. Except for the infections determined by specific pathogens comprised in the TORCH complex, during pregnancy, viral infections express little concern from clinical perspective [210].

#### 6.1.1. Herpes Simplex Virus (HSV)

The mean HSV seroprevalence is 72% in pregnant women, including both HSV-1 and HSV-2 viral infections that result in antibody formation. Throughout the pregnancy, it has been shown that HSV exposure, besides neonatal HSV infection, can also cause spontaneous abortion, preterm labor, and intrauterine growth restriction [211]. To counteract these effects, clinical practice seeks to reduce vertical fetal transmission, reducing the risk of neonatal HSV infection. However, minimal risks of perinatal transmission were registered if antibodies are present at the onset of pregnancy, while it has been shown that there is a risk of 30% to 50% of neonatal infection if the primary infection occurs during late pregnancy [212]. Neonatal HSV infection can be classified into three categories, including localized infection affecting the skin, eyes and mouth (SEM), central nervous system (CNS) infection, and the most severe form, disseminated disease which can cause fatality in 80% of cases, if left untreated [213]. If infected, newborns can exhibit numerous neurologic dysfunctions such as seizures, blindness or learning disabilities. Antiviral treatment in the last month or pregnancy can reduce the frequency of asymptomatic viral shedding, while caesarean delivery is recommended when lesions are present at the onset of labor, in order to reduce the risk of viral transmission even if suppressive therapy was previously used [214].

#### 6.1.2. Varicella Zoster Virus

Varicella zoster virus (VZV) causes an acute, highly contagious disease, commonly referred to as chickenpox, often encountered in childhood. The virus typically incubates for about 15 days and is contagious for 2 days before the onset of the rash until the lesions are crusted and/or cured. After initial infection, the virus may persist latently in the dorsal root ganglia for several years, reactivation of the virus causing herpes zoster, a disease more often encountered in adults [215]. The frequency rate of varicella in pregnancy is 0.7/1000. As varicella is mostly encountered in childhood, many women are already immune before they become pregnant, so that the frequency rate of infection in pregnant women lies between 0.7 and 3 per 1000 cases [216]. However, primary varicella infection during pregnancy is known to cause serious fetal and maternal morbidity and even fetal death. Compared to other childhood illnesses that associate limited and mild symptoms, in the majority of cases, if varicella pneumonia occurs during pregnancy, it can have a much more serious outcome. Statistically, 10% to 20% of pregnant women who contract varicella develop pneumonia, with a death rate of up to 40% [216,217]. On the other hand, fetal morbidity related to congenital varicella syndrome is characterized by intrauterine growth restriction, limb hypoplasia, hydrocephaly, microcephaly, cataracts, and mental retardation. It is believed that the development of this syndrome can be a result of reactivation of the varicella virus in utero, unlike the primary infection of the fetus [218].

#### 6.1.3. Cytomegalovirus

Cytomegalovirus (CMV) is an omnipresent virus that leads to the development of a variety of clinical manifestations, its seroprevalence differing based on geographic area, socioeconomic status and age. Statistically, around 60% of adult women in developed countries are infected with CMV and a greater percentage of almost 90% occurs in women living in developing countries [219]. In order to combat congenital CMV infection, it is important to investigate the maternal CMV antibody positivity. If the infection develops during pregnancy, it can develop numerous comorbidities during pregnancy. Primary maternal infections vary from 1% to 4% of susceptible women and reactivation arises in almost 10% of seropositive women. Regarding the effects on the fetus related to maternal infection, CMV is statistically the most common congenital infection, with an ubiquity of approximately 0.5–2% of live births [220]. CMV affects the lateral ventricles, the organ of Corti and the eighth cranial nerve, thus explaining the frequency of congenital hearing loss among affected individuals. Additionally, human neuronal cells are susceptible to in vitro infection with CMV, which explains the abnormalities in fetal development of the central nervous system [221,222].

#### 6.1.4. Rubella

Rubella is commonly asymptomatic in 25% to 50% of patients. If maternal infection develops in the first trimester, the risk of fetal infection ranges from 50% with a decrease to <1% after 12 weeks, while the risk of congenital rubella syndrome (CRS) is not increased by peripartum maternal infection [223]. Serologic tests must be used in order to diagnose the primary maternal infection. For fetal infection diagnosis, fetal serum IgM detection, and/or amniotic fluid viral culture are advised. Pregnancy abnormalities caused by maternal rubella infection are comprised of fetal infection, fetal growth restriction, stillbirth, CRS, or even spontaneous abortion [224]. CRS represents the neonatal manifestations of antenatal rubella virus infection, and includes, among others, cataract, deafness, neurological, and cardiac defects. However, the risk depends on the gestational age at which maternal infection occurs. Consequently, individualized counseling related to fetal risks and management must be applied [225].

### 6.2. EVs Roles during Antiviral Response

EVs play a role in immune responses by acting as modulators, with antigen-presenting cells derived EVs either activating or modulating immune responses. Moreover, EVs derived from syncytiotrophoblast in nonpathological placenta seem to have an impact in resistance pathways of pathogen infection, and, similar to those from epithelial cells and tumors, they can be either tolerogenic or suppressive [226]. Embryo-derived EVs encapsulate nucleic acids including DNA fragments, microRNA, long non-coding RNA, messenger RNA, and a variety of proteins that can be taken over by cells of the maternal vascular and immune systems and subsequently modulate the pregnancy changes related to maternal physiology [227]. Various studies have demonstrated that the transmission of some viruses to the fibroblast and endothelial compartments is mediated by the miRNA cluster of the chromosome 19, a cluster which is only expressed in the human placenta, overall limiting the infection by induced autophagy in recipient cells [228]. Similarly, Delorme–Axford and colleagues have recently demonstrated the involvement of trophoblast-derived exosomes in impeding viral replication by their upregulating of key proteins involved in the autophagy process, such as light chain 3 (LC3), the ultraviolet irradiation resistance-associated gene (UVRAG) and autophagy related 4C cysteine peptidase (ATG4C) [229]. Furthermore, EVs derived from trophoblast cells have also been speculated to release IFNλ1, thus protecting the fetal compartment from infection caused by pathogens such as the Zika virus [230]. Extracellular vesicles also have a role in the antiviral response by means of transferring nucleic acids. In this way, cells can be reached by signals of viral infection, even when they are not directly infected by the virus, since they can be devoid of the specific receptors [231]. On the other hand, exosomal trafficking can also act in favor of the pathogen, promoting its immune evasion by sheathing it within a secure vesicle, ultimately facilitating the viral spread and the overall infectivity [232,233].

## 7. Preterm Birth

### 7.1. Definition, Etiology, Epidemiology

The World Health Organization estimates that approximately 15 million infants per year are born too early. Preterm delivery is defined as delivery prior to 37 completed weeks of gestation, and is the main cause of neonatal morbidity and mortality [234]. Premature infants, if they survive, are subjected to long-term chronic health problems and even neurological impairment. Regarding maternal consequences, they may vary from cesarean delivery, which subjects the mother to higher obstetrical risks during subsequent pregnancies, to negative psychological outcomes such as anxiety, requiring special attention [235]. Premature birth has multifactorial causes, and for most cases, the precise etiology is still unclear. Some risk factors include: high blood pressure, diabetes, obesity or being underweight, multiple pregnancy, less than six months between pregnancies, previous premature birth, vaginal infections, psychological stress, and tobacco smoking. Furthermore, some studies have also added dyslipidemia and inflammation during pregnancy as contributing factors to premature birth [236]. Clinical events occurring during preterm labor are similar to those in term labor, and consist in increased uterine contractility, dilatation of the cervix, and chorioamniotic membranes rupture. These events are triggered by the switch of the myometrium state from quiescent to intermittent contractions, determined by an imbalance in the pro-inflammatory and anti-inflammatory factors [234]. Among the pro-inflammatory factors there are cytokines such as IL-1 and IL-6, contraction associated proteins including connexin 43 (Cx43), oxytocin receptors (OXT-R), and prostaglandin F2 alpha receptors (PGF2 alpha-R) and chemokines. Uterine quiescence is maintained by progesterone, which represses the expression of these factors. Labor is further promoted by the increasing expressions of the miR-200 family, which favor the catabolism of progesterone and derepresses contractile genes near term [208,237].

### 7.2. EVs in Preterm Birth

One of the indicators of preterm birth (PTB) consists of the isolation and analysis of extracellular vesicles. In a longitudinal study on a group of patients with term and preterm birth, Menon et al. have observed a total of 173 miRNAs with notable changes in circulating exosomes across three gestational periods by using next-generation sequencing. The altered miRNAs could be separated into several groups with different change tendencies over time, essentially showing that the miRNA content of maternal exosomes could be used as a biomolecular identification method of pregnancy progression [238]. Furthermore, a phenomenon associated with infections that can lead to PTB is hyperresponsiveness to low concentrations of bacterial endotoxin, and it can, at least in theory, be predicted with the aid of exosomes. Certain infections can modify the function of pattern recognition receptors (PRRs) at molecular level, including Toll-like receptors (TLRs), both in quality and quantity. In this regard, it has been demonstrated that placental type I IFN β was downregulated by the virus, followed by intrinsic TLR4 mediated proinflammatory cytokines regulation in the trophoblast and enabling its cells to create an endotoxin response. Judging by these findings, it can be deduced that multiple infection-related pregnancy complications are of polymicrobial nature, evolving from an initial infection. The first stage of the infection is viral, which negatively impacts the PRRs’ response, thus facilitating a second stage of the infection, bacterial. The cumulative action of these pathogens can induce preterm delivery by causing a dysregulated inflammatory response [239]. Additional studies, such as the one conducted by Menon and colleagues, have looked at groups of patients including term not in labor, term in labor, preterm premature rupture of membranes, and PTB patients, comparing the proteomes of maternal plasma exosomes. Menon et al. identified 72 proteins that displayed notable changes among these four groups, leading to the hypothesis that the main instigator of PTB relates to hematological dysfunctions rather than inflammation, which instead occurs as a result of functional changes such as complement activation, acute phase signaling, liver X receptor (LXR)/ retinoid X receptor (RXR) activation [240]. In a similar manner, Cantonwine et al. focused their study on a group of women ranging between 10 and 12 weeks of gestation who developed spontaneous PTB at 34 weeks, at which time they analyzed circulating MVs. A total of 62 proteins qualified for diagnosis from 132 proteins evaluated through ROC analysis, of which a group of three exosomal proteins, namely alpha-2-macroglobulin (α2M), human endogenous medium-reiteration-frequency-family-34 ORF (HEMO), and mannose binding lectin 2 (MBL2), displayed a specificity of 83% with median AUC of 0.89. This group, if validated, would enable the stratification of patients which are subject to a high risk of spontaneous PTB before clinical symptoms appear [241].

## 8. Fetal Growth Restriction

### 8.1. Definition, Etiology, Epidemiology

Fetal growth restriction (FGR) takes place when the fetus is unable to reach its intrauterine growth potential and frequently occurs as a result of placental malfunctions. It is currently considered that fetuses with FGR have a greater risk of developing long-term health defects, morbidity, and even mortality. After birth they can suffer from impaired neurological and cognitive development and, in adulthood, there are high chances of developing cardiovascular or endocrine diseases [242]. Statistically, FGR occurs in 5–10% of total pregnancies, and it is worldwide considered as the first cause of perinatal mortality. Moreover, it is also thought to be the main cause of premature birth and intrapartum asphyxia [243]. Currently, there is no worldwide standard for FGR diagnosis. In clinical practice, FGR is diagnosed by using a statistical deviation from the fetal size having a population of reference. The percentile limits are 10, 5, or 3. However, these limits are considered to indicate if a fetus is small for gestational age (SGA). As opposed to fetuses that are FGR positive, SGA fetuses include healthy ones with low risk for abnormal perinatal outcomes, based on a rather small physical constitution. On average, 70% of fetuses are considered weighing below the 10th percentile due to inherited physical factors and parent ethnicity [244]. There are multiple factors involved in the etiology of FGR, and they are typically divided into maternal and fetal causes that result in uteroplacental vascular insufficiency. Fetal factors include chromosomal abnormalities such as trisomy 13, 18, and 21, in particular, genetic syndromes such as mutations in the gene responsible for insulin-like growth factor production, intrauterine infections, especially with certain viruses that can cause placentitis, multiple gestation pregnancies, inborn errors of metabolism. Maternal factors on the other hand generally include clinical diseases, all types of hypertensive pregnancy diseases, insulin-dependent diabetes mellitus with vasculopathy, autoimmune diseases, alcohol and drug use, smoking, and nutritional disorders including chronic malnutrition. Uteroplacental factors refer to structural abnormalities, inadequate placentation, which refers to the existence of an area with high resistance to blood flow that results in decreased nutrition of the intervillous space, and changes in placental implantation and attachment [245]. At this point, there is no treatment that can reverse or stop placental insufficiency, with strategies in the management of the fetus suffering from FGR including evaluating its vitality and planning when the delivery should take place [246].

### 8.2. EVs in Fetal Growth Restriction

As studies have shown that EVs have specific roles in the communication between the endometrium and the embryo, they also promote proinflammatory cytokine release with an important role in altering inflammatory responses during pregnancy by suppressing natural killer cells and macrophages activation [247]. In a group study conducted by Miranda et al. targeting women who gave birth to small fetuses, concentrations of total and placental EVs in circulation were detected, using CD63 and PLAP labelling. PLAP+ CD63+ exosomes to PLAP− CD63+ ratio was used to show the contribution of relative placental exosomes to the total. A trend according to the severity of the disease could be deduced by correlating the above ratio with the percentile of birth weight. Therefore, the relative concentration of placental EVs could act as a fetal growth marker [86]. Another study conducted by Rodosthenous et al. reported higher expression levels of miR-942-5p, miR-223-5p, miR-20b-5p, miR-324-3p, and miR127-3p in second trimester patients that later gave birth to SGA infants. Of these, miR-127-3p was also linked to abnormal fetal growth [241,248].

## 9. Congenital Anomalies

Congenital anomalies refer to the existence of some defects which manifest as structural or functional abnormalities that occur during intrauterine development and are detected either prenatally using current imaging techniques and / or molecular biology techniques, at birth or later in childhood [249]. Although almost half of all congenital anomalies cannot be correlated with one single specific cause, there is evidence pointing to specific risk factors linked to the occurrence of congenital anomalies, with genetic aberrations and gene mutations playing important roles in this regard. Other important risk factors refer to socioeconomic and demographic factors, environmental factors and maternal infections during pregnancy [250]. Around 6% of babies worldwide are affected by these anomalies, which could lead to long-term disabilities, chronic illness or even infant and/ or childhood death [251]. While some congenital abnormalities such as hernias, cleft lip and cleft palate, or foot deformities can quite easily be treated by means of surgery, more complex defects—such as cystic fibrosis, thalassemia, and hemophilia—can only be managed using non-surgical options with less favorable results. Together with the most common and severe birth defects, including heart defects, neural tube defects, and chromosomal aneuploidies, congenital anomalies are one of the main causes of global burden of disease, posing significant impact not only on the individual and their family, but also on the health care system and society [252]. For this reason, several studies have been conducted in recent decades on the assessment of causality and clinical presentation of major birth defects, in which the biological significance of extracellular vesicles as mediators of intercellular signaling, especially in embryo-fetal/maternal communication through the placenta, has been the main subject. With the development of molecular diagnostic techniques, the possibility to study specific EVs has led to the discovery of biomarkers and new methods that can be used for early diagnosis, prevention, and prognosis of pathologies associated with pregnancy [253].

### 9.1. Down Syndrome

Down syndrome (DS) is a chromosomal condition affecting around 1 in 800 births worldwide, caused by human chromosome 21 (Hsa21) trisomy, and it is primarily associated with intellectual disability and neurodevelopmental abnormalities occurring early in the embryonic life [254,255]. Being the most common chromosomal disorder, intense studies into its causes have been carried out, however, variations in clinical manifestations have been correlated with the involvement of epigenetic factors [256]. In about 95% of cases, the genetic mechanism to blame revolves around the process of meiotic nondisjunction that results in the occurrence of 47 chromosomes. Other mechanisms involved refer to the Robertsonian translocation of an additional chromosome 21 to chromosomes 13, 14, 15, or 22, or the duplication of a delimited segment of chromosome 21 [257]. In order to better comprehend the processes that stand at the basis of DS, numerous studies have been conducted, postulating the idea that both neural-derived and non-neuronal EVs contribute to its development, due to their ability to facilitate intercellular communication at the level of the CNS [258]. As the composition of the CNS broadly consists of several types of cells with distinct morphologies and functions, EVs may contain different loads depending on their origin. Through their cargo, they are capable of modulating signal transduction pathways, leading to subsequent changes in neurogenesis, gliogenesis, synaptogenesis, and the formation of network circuits, as well as myelination and synaptic pruning [254]. Several studies have shown that in the case of CNS dysfunctions, including DS, the composition of EVs varies depending upon biogenetic pathways and parental cells [259]. In addition to the endosomal sorting complexes required for transport (ESCRT), apoptosis-linked gene 2-interacting protein X (Alix), and tumor susceptibility gene 101 (TSG101) proteins, currently considered standard markers of exosomes, regardless of parental cells, several types of membrane, cytosolic, and cytoskeletal proteins have commonly been found in exosomes isolated from neuronal cells [254,260,261]. In the interest of facilitating non-invasive prenatal testing, Erturk et al. have analyzed miRNAs originating in trophoblast-derived EVs from pregnant women with fetuses suffering from DS, and found that miR-99a and miR-3156 levels were significantly higher compared to women carrying healthy fetuses [262]. Similarly, in a proof-of-concept study using high-throughput quantitative PCR (HT-qPCR) on samples from DS pregnancies and controls, Kamhieh-Milz et al. managed to identify 36 mature miRNAs notably differentially expressed in affected pregnancies, emphasizing their potential as stable biomarkers [263]. Similarly, using microarray-based genome-wide expression profiling, Lim et al. analyzed placental samples from euploid and DS fetuses, and found that miR-3196 and miR-1973 had higher expression levels in placentas from affected fetuses [264].

### 9.2. Neural Tube Defects

Neural tube defects (NTDs) make up the second most common group of congenital anomalies involving the CNS, with a prevalence of 1/1000 newborns worldwide. They primarily occur as a result of a defective neural tube closure during neurulation in early embryonic development [265]. Manifestations of this abnormality vary depending on location, from cranial to more caudal regions, and include exencephaly, which eventually leads to anencephaly, encephalocele, spina bifida, and myelomeningocele, among others [266]. Although the pathogenesis of NDT is not yet fully elucidated, it has been shown to be multifactorial, involving both genetic and epigenetic factors [267]. Numerous signaling pathways and genes that facilitate cell-to-cell communication are required for succesful neural tube closure, including the wingless/integrated (Wnt) pathway, bone morphogenetic protein (BMP) signaling pathway, Sonic hedgehog (Shh) signaling pathway [268], along with genes linked to neural patterning, growth, and cell intercalation, such as axis inhibition protein 2 (AXIN2), lymphoid enhancer-binding factor 1 (LEF1), and paired box gene 3 (PAX3) [269]. Epigenetic modifications of histones have also been reported, as well as DNA methylation and aberrant metabolism of folate and related enzymes [270]. In a recent study involving a fetal lamb model of spina bifida, Kumar and colleagues have analyzed the secretome of placenta-derived mesenchymal stromal cells (PMSC), and found that PMSC-derived exosomes could mend limb paralysis through their cargo, found to be rich in RNAs and proteins associated with neuronal survival, such as galectin 1, thus highlighting their neuroprotective function [271]. In a similar manner, Gu et al. have looked at the placental miRNA profiles of NTD pregnancies and, after analyzing a panel of 887 human miRNAs, found six upregulated miRNAs unique to these pregnancies [272].

### 9.3. Congenital Heart Defects

The most common birth defects and the leading cause of infant death from birth defects, with an incidence of 8/ 1000 newborns, are congenital heart defects (CHDs). These circulatory system abnormalities can be attributed in approximately 20% of cases to genetic syndromes—including trisomies 13, 18, 21, and Turner syndrome—along with submicroscopic deletions or duplications [273]. Despite abundant studies, the exact genetic cause of these abnormalities has not been fully elucidated, but it has been shown that the etiology of CHD is multifactorial, resulting from a constellation of non-coding genetic, epigenetic and environmental factors [5]. Following extensive population-based studies carried out in recent decades, it has been found that CHD pregnancies are associated with an increased risk of developing pathologies linked with placental disease. This may be due to the fact that, as vascular organs, both the heart and the placenta develop simultaneously, thus potentially sharing common developmental pathways [274]. Studies published to date on CVDs and the involvement of EVs in their occurrence do not provide a clear picture of the molecular mechanisms and communication pathways involved in the etiology of these syndromes. EVs are known to be involved in placental-related physiological and pathological processes such as inflammation and vascular calcification, but they also perform regulatory functions in the cardiovascular system that are related to thrombosis and vasoactive reactions [5]. To this extent, as Jia and colleagues have demonstrated, maternal and umbilical exosomes aid both angiogenesis by facilitating the proliferation and migration of human umbilical vein endothelial cells (HUVECs), and the formation of the heart tube, through their bioactive cargo. Specifically, they found a set of upregulated miRNAs present in both types of EVs, which they could link to the modulation of cell migration and proliferation [80]. Similarly, using microarray followed by RT-PCR, Gu et al. have recently observed the miRNA expression of CHD pregnancies, and, based on an elevated statistical significance level, found a panel of four miRNAs consistently dysregulated among CHD pregnancies, supporting the potential role of exosome-miRNAs as non-invasive biomarkers for CHDs [275].

## 10. Conclusions

The placenta is an ultra-specialized organ that establishes the connection between the carrier mother and the developing fetus, and extracellular vesicles assist the intricated interaction between the two organisms. Due to their unique molecular composition, placenta-derived EVs are able to perform several functions, especially the transfer of molecular signals between the fetus and mother, and any disturbances that may affect these molecules can contribute to the occurrence of various pregnancy-related disorders. To date, the involvement of these specialized messengers has been demonstrated both in normal and pathological aspects of pregnancy, ranging from embryo implantation, vascular remodeling and parturition, to maternal systemic inflammation, gestational hypertension and diabetes mellitus, as well as preterm birth due to various pregnancy related conditions and congenital anomalies. Due to the possibility to detect EVs and their contents in the peripheral blood of women starting in early pregnancy, they can be used as timely prediction or diagnostic biomarkers of pregnancy complications and fetal developmental disorders. However, due to the lack of an established standardized detection method, the analysis of placental EVs has yet to reach the end goal of clinical application. Nevertheless, their non-invasiveness and accuracy prompt researchers to welcome further empirical evidence and experimental studies to validate the feasibility and clinical applicability of EVs as circulating predictive biomarkers and, in time, EV-based therapies.

## Figures and Tables

**Table 1:** Markers that confirm the presence of EVs.

Category I	Category II	Category III
GPI-anchored or transmembrane proteins, demonstrating the lipid bilayer of the EV	Cytosolic proteins in eukaryotic cells and Gram-positive bacteria Periplasmic proteins in Gram-negative bacteria	Constituents of non-EV factors that help evaluate the degree of contamination of the sample (e.g., APOA1/2, APOB, albumin, UMOD)

GPI: glycosylphosphatidylinositol; APOA1/2, APOB: apolipoproteins A1/2 and B; UMOD: uromodulin.

**Table 2:** Isolation methods used in the detection of pregnancy-related EVs.

Biological Sample	Potential Interfering Factors	Isolation, Separation and Concentration Techniques	Characteristics	References
Plasma/serum of pregnant women	Pre-/postprandial status Medication Sample volume Container type Processing time Choice of anticoagulant	Differential centrifugation Sequential centrifugation Ultracentrifugation Ultrafiltration	Standard protocol for EVs isolation from biological fluids. Requires additional steps for purification of pregnancy-associated EVs from other vesicles and co-isolated proteins	[38,39,40]
Fluid from cultured placental tissue explants Syncytiotrophoblast	Specific infectious and noninfectious diseases Technical factors Storage and processing	Differential centrifugations Ultracentrifugation Ultrafiltration Chromatographic/immunosorbent procedure Size exclusion chromatography Precipitation Immunoaffinity-based capture	Used to study the composition and biological roles of placental EVs in normal and pathological pregnancies. Requires ex vivo cultures of placental explants at different gestational ages. Techniques for biological samples collection may damage the products of conception	[38,41,42]
Placental perfusate Placental homogenate	Specific infectious and noninfectious diseases Technical factors Storage and processing	Differential centrifugation Gel filtration Ultrafiltration Affinity chromatography Microfluidic technology	Biological products accessible only after delivery Enrichment of EV preparations requires purification steps	[38,43,44,45]

**Table 3:** Methods for the detection and confirmation of pregnancy-related EVs.

Detection/Confirmation Techniques	Characteristics	References
Flow cytometry	Not selective enough to analyze membrane and non-membrane structures, as it detects all particles with CD81, CD9, and CD63 markers It is recommended to use appropriate negative controls (antibodies, isotype controls, etc.)	[36,37,38,46]
Western blot	Most commonly used technique, although not selective enough Employs the use of STB marker PLAP, as well as exosomal markers Alix and CD63, and potential contaminating markers such as platelet, red blood cell, and leucocyte markers	[37,38,46]
Fluorescence nanoparticle tracking analysis (fl-NTA)	Typically used to determine PLAP-positive EVs, thus reliably identifying STBMV Facilitates counting, sizing, and phenotyping of EVs	[47]
Imaging techniques such as electron microscopy, immunoelectron microscopy, scanning electron microscope (SEM), transmission electron microscope (TEM), cryogenic electron microscopy (cryo-EM), scanning probe microscopy (SPM), atomic force microscopy (AFM), super-resolution microscopy (SRM)	Used to visualize single EVs at high resolution, providing information on their structure and composition, especially when combined with antibody-mediated detection of EVs components Allows the detection of exosomal markers directly on the nanovesicle surface These techniques are not interchangeable as they do not offer information about EVs of comparable characteristics When dealing with insufficiently purified preparations, co-isolated impurities can lead to misinterpretation of biochemical contents	[38,48,49,50,51]

**Table 4:** Role of placental EVs during different pregnancy states.

Pregnancy State	Role of Placental Extracellular Vesicles
Normal pregnancy	Promotion of embryonic implantation and placental development Maternal immune response modulation Induction of fetal vasculogenesis Induction of inflammatory response during parturition
Systemic inflammation	Induction of inflammation through altered cargo composition Increased inclusion of inflammation inducing agents, such as HMG nuclear proteins, TNF-α, GM-CSF, IFN-γ, IL-6, IL-8, miR-155, miR-494, miR-181a, and miR-210 Diminished inclusion of anti-inflammatory agents, such as miR-548c-5p
Gestational hypertension	Increased release during PE Induction of MSIR
Gestational diabetes mellitus	Increased release during GDM Increased inclusion of IR implicated miRNAs, such as hsa-miR-125a-3p, hsa-miR-99b-5p, hsa-miR-197-3p, hsa-miR-22-3p and hsa-miR-224-5p Differential inclusion of miRNAs, such as miR-16-5p, miR-17-5p, and miR-20a-5p
Viral infections	Inclusion of anti-viral agents, such as LC3, UVRAG, ATG4C, and IFN-λ1 Speculated beneficial role for viral spread through immune evasion Viral sheathing by exosomes
Fetal growth restriction	Increased ratio of placental to total exosomes Increased inclusion of miRNAs, such as miR-942-5p, miR-223-5p, miR-20b-5p, miR-324-3p, and miR-127-3p

HMG: high mobility group; TNF-α: tumor necrosis factor α; GM-CSF: granulocyte-macrophage colony-stimulating factor; IFN-γ: interferon γ; IL: interleukin; PE: preeclampsia; MSIR: maternal systemic inflammatory response; GDM: gestational diabetes mellitus; IR: insulin resistance; LC3: microtubule-associated protein 1 light chain 3; UVRAG: UV radiation resistance-associated gene; ATG4C: autophagy related 4C cysteine peptidase.

**Table 5:** Diagnostic criteria for hypertension.

Organization	Number of Measure-Ments	Hyper-Tension	Mild	Moderate	Severe	Emergent
ACOG, 2019 [161]	2 (minimum 4 h apart)	SBP ≥ 140 mmHg and/or DBP ≥ 90 mmHg	-	-	SBP ≥ 160 mmHg and/or DBP ≥ 110 mmHg	-
Regitz-Zagrosek, V.; et al. ESC, 2018 [162]	-		SBP ≥ 140–159 and DBP ≥ 90–109 mmHg	-	SBP ≥ 160 mmHg or DBP ≥ 110 mmHg	SBP ≥ 170 mmHg or DBP ≥ 110 mmHg
Magee, L.A.; et al. SOGC, 2014 [163]	2 (minimum 15 min apart)		-	-	SBP ≥ 160 mmHg and/or DBP ≥ 110 mmHg	-
Redman, C.W. RCOG, 2011 [164]	-		SBP ≥ 140–149 and DBP ≥ 90–99 mmHg	SBP ≥ 150–159 and DBP ≥ 100–109 mmHg	SBP ≥ 160 and DBP ≥ 110 mmHg	-

Diagnostic thresholds for the classification of hypertension as mild, moderate, severe or emergent. ACOG: American College of Obstetricians and Gynecologists, ESC: European Society of Cardiology, SOGC: Society of Obstetricians and Gynecologists of Canada, ISSHP: International Society for the Study of Hypertension in Pregnancy, RCOG: Royal College of Obstetricians and Gynecologists.

**Table 6:** Diagnostic criteria for GDM.

Organization	Year	Fasting (mg/dL)	1 h (mg/dL)	2 h (mg/dL)
ADA	2018 [191]	95	180	155
ADIPS	2014 [192]	92	180	153
FIGO	2015 [188]	92	180	153
WHO	1998 [193]	126	–	140
WHO	2013 [187]	92	180	153
IADPSG	2010 [190]	92	180	153

Glucose concentration thresholds in serum, above which a hyperglycemia is considered overt diabetes. The diagnostic Oral Glucose Tolerance Test (OGTT) is performed during a fasting period and after injection of 75 g of glucose (after 1 and 2 h). ADA: American Diabetes Association, ADIPS: Australasian Diabetes in Pregnancy Society, FIGO: International Federation of Gynecology and Obstetrics, WHO: World Health Organization, IADPSG: International Association of the Diabetes and Pregnancy Study Groups.
